# A Delphi Study to prioritize implementation strategies to increase the adoption of screening and referral for brain injury among survivors of intimate partner violence and sexual assault within community-based organizations

**DOI:** 10.21203/rs.3.rs-6925920/v1

**Published:** 2025-07-15

**Authors:** Shireen S. Rajaram, Emiliane Lemos Pereira, Kathy Chiou, Peggy Reisher, Christopher Wichman, Megan N. Miller, Paul A. Estabrooks

**Affiliations:** University of Nebraska Medical Center; University of Nebraska Medical Center; University of Nebraska–Lincoln; Brain Injury Association of Nebraska; University of Nebraska Medical Center; University of Utah; University of Utah

**Keywords:** brain injury, screening, intimate partner violence, domestic violence, community-based organizations

## Abstract

**Background:**

Women who experience intimate partner violence (IPV) are at a high risk for injuries to the head, neck, and face that can result in a traumatic brain injury (BI). Despite increasing evidence of the high risk for BI in this vulnerable population, BI screenings remain critically under-implemented in community-based organizations (CBOs) serving IPV survivors. The aim of this community-engaged dissemination and implementation project was to co-identify implementation strategies to increase the adoption of brain injury (BI) screening and referral within intimate partner violence (IPV)-serving CBOs.

**Methods:**

We used a modified Delphi method to prioritize 47 CBO relevant strategies from the Expert Recommendations for Implementation Change compendium to increase the adoption of BI screening and referral among IPV-serving CBOs. In Round-1, 14 Community-campus advisory board (CAB) members, including representatives from 10 CBOs prioritized relevant strategies in a virtual meeting. In Round-2, 62 CBOs staff members responded to a survey to refine a subset of prioritized strategies to 6–8 primary strategies that could be tested across the CBOs.

**Results:**

CAB participants identified 21 strategies as particularly relevant to CBOs including 4 educational, 2 technical assistance, 5 staff and leadership, 4 management and evaluation, and 6 organizational workflow strategies. Survey responses indicated rated 7 of the 21 strategies were most consistently rated as relevant and feasible. The final list of 7 strategies included training opportunities, ongoing consultation, developing implementation plans, establishing local screening and referral protocols, soliciting survivor feedback, promoting adaptability, and tailoring strategies to CBOs contexts.

**Conclusions:**

This study highlights the importance of creating tailored implementation strategies within IPV-serving CBOs to enhance the adoption of brain injury screening and referral protocols. The identified strategies offer valuable insights into optimizing support for IPV survivors and advancing public health interventions.

## Background

Intimate partner violence (IPV) is a serious public health issue that affects millions of people in the U.S and worldwide. IPV is defined as violence or aggression that occurs within a close relationship, including with a current or former spouse, or a dating or sexual partner. Violence or aggression can include physical violence, sexual violence, stalking, and psychological abuse [1–3]. Statistics reveal that 1 in 2 women and 1 in 4 men have experienced sexual violence, physical violence, or stalking by an intimate partner in their lifetime and reported an IPV-related impact, including injury, fear, safety concerns, and/or need for supportive services [3]. While IPV affects all communities, it disproportionally affects women and racial/ethnic minorities [3].

IPV also extends beyond the immediate trauma to inflict profound and often hidden injuries, particularly on women. Studies have highlighted that women who experience IPV are at a high risk for injuries to the head, neck, and face that can result in a traumatic brain injury (TBI) [4–8]. These IPV-related TBIs can have lasting effects, leading to chronic symptoms such as headaches, memory and cognitive issues, concentration problems, dizziness and anxiety[9–14]. IPV survivors are also at high risk for an Acquired Brain Injury (ABI) resulting from strangulation that involves hypoxia and anoxia when the oxygen supply to the brain is restricted or completely cut off [4, 5, 15].

IPV-related brain injury (BI), which includes TBIs and ABIs is likely to be repeated, and women may experience multiple hits to the head in a short span of time [10, 12, 16]. A study by Rajaram and colleagues (2020), showed that of 171 IPV survivors screened, 92% experienced a hit to the head or strangulation [17]. Moreover, 52% reported that it occurred more than four times in their lives. People who experience repeated brain injuries before the brain recovers from the initial trauma are at high risk for neurological deficits, depression, suicide, and Alzheimer-like symptoms [18, 19].

IPV survivors may not seek medical care following a BI. They may not trust medical providers [20, 21] or have access or the autonomy to seek medical help for their injuries [9, 22]. They may also be concerned about shame, fear, and stigma associated with IPV [23, 24], and retaliation from their abusers resulting from the disclosure of abuse [25]. IPV-related BIs are hidden and, often, have no visible signs of physical trauma; thus these injuries can be missed or misdiagnosed by healthcare and social service providers [15, 16, 26], and may be minimized by survivors [27]. One study [28] showed that only 35% of IPV survivors who were at high risk for BI received medical treatment. We also found that IPV survivors may be more likely to reach out to community-based organizations (CBOs) that provide social services to survivors of IPV, sexual assault, and sex trafficking.

Despite increasing evidence of the high risk for BI in this vulnerable population[4–6, 10, 29–31], there remains an inadequate focus on BI and BI screening among IPV survivors [12, 32]. As BI continues to go unreported, undetected or misdiagnosed by mainstream healthcare and social service providers, other avenues, such as front line workers or CBOs, whom survivors may be more willing to disclose their needs to, have a unique opportunity to assist in the identification of a potential BI. Thus, it is imperative that IPV-serving CBOs integrate routine screening for possible BI and connect survivors to referral services, where evidence-based BI interventions may be available and accessible.

Integrating BI screening approaches and referrals that are specifically tailored to IPV survivors holds great potential benefits. For example, a qualitative study [33] on screening for BI with a follow-up neuropsychological evaluation (NPE) showed the potential to be acceptable to IPV survivors and service providers. Understanding the facilitators and barriers to the use of evidence-based interventions by IPV-serving CBOs will help increase the chances for improved recovery through appropriate neurorehabilitation, particularly for disenfranchised groups who may lack the means to access, or have low trust in, medical care.

Unfortunately, little is known about implementation strategies for screening by IPV-serving CBOs or how these organizations integrate screening and provide timely referrals – in routine practice – to aid women in the recovery process. A recent study in behavioral health settings found that only 25% of providers adopted BI screening post-training and suggested that implementation strategies that include additional training, leadership engagement, and state mandates may be needed to support widespread systematic uptake of BI screening [34]. This aligns with the Expert Recommendations for Implementing Change (ERIC) project that highlighted a range of strategies to support, primarily healthcare, implementation of evidence-based interventions [35]. That project identified 73 unique strategies that can be characterized as those that focus on education and training, staff and leadership activities, technical assistance, management and evaluation, and organization and workflow factors. However, outside of providing training, to date, no known studies have used these expert recommendations to improve the dissemination and implementation of BI screening and referrals to service provision among IPV-serving CBOs (or other behavioral health settings). Furthermore, while many CBOs participate in community-engaged research approaches, there is also a gap in understanding how community-engaged dissemination and implementation (CEDI) strategies [36] may be applied collaboratively with IPV-serving CBOs. This study aimed to co-identify appropriate BI screening and referral implementation strategies with IPV-serving CBOs and to develop guidance for broad adoption, implementation, and sustainment.

## Methods

### Setting and participants.

This study used a community-academic partnership approach that involved a team of research scientists, a project manager, a non-profit community partner, and a community advisory board (CAB). Collaboratively, the project team identified 12 community-based programs (10 CBOs) from both rural and urban areas in a Midwestern state that served survivors of IPV and sexual assault to participate in a larger project focused on increasing BI screening and referral strategy implementation for women experiencing IPV. All CBOs involved in the project were participants in the study’s CAB and expressed an interest in conducting BI screening and referrals for IPV survivors who had used their services.

Members of the CAB (n=14) participated in the modified Delphi process [37] described below and provided background information including organizational affiliation, role, profession, experience, demographics, and racial/ethnic backgrounds. This study was approved by the Institutional Review Board (IRB) at the University of Nebraska Medical Center (UNMC) under protocol number 0259–23-EX. Consent to participate was obtained from all participants. A summary of participants’ characteristics is in Table 1.

### Theoretical Approach

The ERIC strategy list was reviewed through the lens of constructs from the Practical, Robust Implementation and Sustainability Model (PRISM) to ensure coverage of determinants at the organizational (e.g., leadership support, workflow fit), recipient (e.g., survivor readiness, staff training needs), external (e.g., funding or policy mandates), and infrastructure (e.g., availability of technical assistance) levels. This mapping informed the initial CAB discussions and selection process for the Round 1 Delphi.

### Strategy identification process.

During the initial planning phase, spanning two and a half months, community partners and researchers engaged in discussions and consensus-building to create a prioritized list of 47 possible ERIC evidence-based strategies from an initial pool of 73 strategies [35]. This process involved an iterative multi-round approach [37, 38], wherein the core team, including experts in dissemination and implementation science (PE and EP), as well as in intimate partner violence (SSR), closely examined the 73 strategies outlined in ERIC [35] (see Table 2).

### Modified Delphi Approach.

A modified Delphi method was used to determine the most applicable strategies for increasing the adoption of BI screening and referral protocols among IPV-serving CBOs [37, 38]. We selected a modified Delphi technique due to its structured process for achieving expert consensus on priorities in areas where empirical evidence is limited and practice contexts are highly variable (McMillan 2016; Boulkedid 2011). This approach enabled broad participation across diverse CBOs and allowed us to systematically refine strategy priorities using community-engaged principles aligned with implementation science best practices. The Delphi process can be used to gather input virtually from a diverse group of people while maintaining anonymity which facilitates engagement and participation across different levels of an organization [37, 38]. Our approach aligns with other Delphi applications in implementation science [39, 40] by emphasizing context-sensitive strategy tailoring and extends this approach to CBOs providing IPV services. We constructed a QUALTRICS survey (see Appendix for survey questions) and organized the 47 ERIC-derived implementation strategies into five distinct categories: educational and training strategies, technical assistance strategies, staff and leadership development strategies, quality management strategies, and integration into organizational workflow.

#### Delphi – Round 1

In the initial round of the Delphi process, we conducted an interactive consensus-building video-conference meeting with CAB members. A total of 14 CAB participants representing the 12 community-based programs and 4 research team members (SSR, PE, EP, CW) attended the meeting. First, participants were divided into three virtual sub-groups, and each group was assigned a subset of two ERIC categories. Moderators (SSR, PE, EP) facilitated discussions within each group, explained the categories, reviewed and clarified strategy definitions, and presented a rating process for participants to complete individually. Participants then rated around 17 strategies per group using a response set of “must-have,” “nice-to-have,” “don’t need,” or “need more information” as the strategy related to improving the uptake of BI screening and referral within their respective organizations. Second, CAB members reconvened as a whole group to discuss the results from Round 1 ratings. The video conference moderator (EP) presented preliminary results, particularly focusing on strategies rated as “must-have” and those needing further clarification. Third, following the CAB meeting, the research team reviewed the results and refined the list of ERIC strategies based on participant ratings and discussions [35].

#### Delphi – Round 2

In Round 2 of the Delphi process, a QUALTRICS electronic survey included 21 ERIC-derived implementation strategies that were rated highest during Round 1. The strategies were organized into five distinct categories: 1) educational and training strategies; 2) technical assistance strategies; 3) staff and leadership development strategies; 4) quality management strategies; and 5) integration into organizational workflows.

The survey was distributed via email to staff across the 12 CBO programs and respondents were instructed to rate each of the 21 strategies based on the predefined criteria: “must-have,” “nice-to-have,” “don’t need,” or “need more information.” Emails were sent to the CBOs liaisons requesting that they distribute the survey to all staff members. Two emails were sent to all CBOs with a 3^rd^ targeted reminder to CBOs with low response rates.

### Analyses.

Round 1 survey responses were used to reduce the number of strategies reviewed by CBOs staff. The initial step included combining similar strategies based on CAB feedback. Consistent with recommendations for Delphi reporting (Diamond 2014), consensus for each stage was operationalized as ≥75% agreement across respondents, which served as the basis for final strategy selection. That is, strategies were included in Round 2 if they were: 1) designated as high-priority (“must-have”) by at least 75% of participants or 2) strategies consistently regarded as a combination of high- (“must-have”) or moderate-priority (“nice-to-have”) by 75% of participants. This analysis resulted in a list of 21 strategies for review by CBOs staff in Round 2. Round 2 used the same process of inclusion of strategies (i.e., 75%) based on CBOs staff perceptions’ of potential effectiveness and relevance, resulting in the selection of 7 strategies for inclusion in the final list.

## Results

The initial review of the ERIC strategies led to the removal of 26 strategies that were perceived as either irrelevant to the project (n=17) or redundant with other strategies that had been co-developed by our community-academic partnership as part of the study protocol (n=9). Subsequently, the remaining 47 strategy definitions were collaboratively adapted to better align with the multi-level perceptions and characteristics of BI screening recipients, CBOs implementation infrastructure, and potential external environmental factors that could influence BI screening reach, adoption, implementation, and maintenance in CBOs (see Table 2).

### Round 1

Fourteen CAB participants actively engaged in the Zoom interactive meeting, representing all 12 programs. Most participants (43%) were between 36–45 years old, all identified as female, with educational backgrounds ranging from college (29%) to postgraduate (29%).

Of the 47 strategies reviewed, the CAB participants identified 21 strategies with a high level of agreement. Following our established statistical criterion—selecting strategies identified as a high priority by at least 75% of participants or as a moderate priority by 75% of participants—the following strategies were selected for inclusion in round 2: four from educational strategies (numbers 2, 4, 11, and 12); two from technical assistance (numbers 16 and 17); five related to building staff and leadership (numbers 18, 19, 20, 23, and 26); four concerning quality management (numbers 27, 28, 31, and 32); and six focused on organizational workflow (numbers 37, 38, 41, 42, 43, and 45). See table 3 for the list of strategies with the percentage of agreement (see Table 3).

Strategies that were identified as similar included “Create locally tailored educational materials that explain the purpose and process of brain injury screening” (prioritized by 67% of participants; Strategy 7), and “Use engaging and interactive training methods that encourage active participation and knowledge retention” (prioritized by 78%; Strategy 9) and “Offer continuous training opportunities to staff members to ensure they remain competent in conducting brain injury screenings” (prioritized by 78%; Strategy 4). These strategies were merged, and the strategy was rephrased as follows: “Offer continuous training opportunities to staff members, including the creation of locally tailored educational materials and the use of engaging, interactive training methods to ensure they remain competent in conducting brain injury screenings”. See Table 4 for strategies included in Round 2.

### Round 2

Round 2 surveys were distributed to CBOs staff members (n=140). Sixty-two community partners completed an online survey that was distributed by email. Among them, 29% were aged between 36–45 years, 90% identified as female, and their educational backgrounds varied from college (29%) to postgraduate (26%).

Of the 21 strategies considered, seven were consistently rated as high priority. Following our predetermined statistical criteria, selecting strategies identified as a high priority by at least 75% of participants or as a moderate priority by 75% of participants, the selected strategies included one educational strategy (#4); one technical assistance strategy (#17); one staff and leadership strategy (#19); two quality management strategies (#28, 31); and two organizational workflow strategies (#37, #38). See Table 5 for the final list of strategies. The final set of seven strategies are also aligned with PRISM constructs. Several strategies—such as providing training opportunities and developing formal implementation plans—address organizational characteristics by building staff capacity and creating structured processes to support adoption. Ongoing consultation and the establishment of local screening and referral protocols align with PRISM’s implementation and sustainability infrastructure domain, offering ongoing support and systematized procedures that promote routinization. Strategies like soliciting survivor feedback and promoting adaptability emphasize recipient characteristics and the importance of tailoring services to meet the needs, values, and preferences of those receiving care. Finally, tailoring strategies to CBO context represents a cross-cutting application of PRISM, underscoring the need to accommodate organizational culture, external demands, and contextual realities. Together, these findings highlight how co-identified strategies successfully addressed multilevel PRISM determinants, reinforcing its utility as a guiding framework for implementation strategy selection in community-based settings.

## Discussion

Our study extends previous dissemination and implementation science research by adding to modified Delphi techniques focused on system-level policy[39] or evidence grading[40] to the use of a Delphi process aimed to co-prioritize actionable strategies that align with the practice realities of under-resourced CBOs serving survivors of IPV. To the best of our knowledge, this is the first study to apply co-identification or ERIC strategies [35] in partnership with CBOs to increase the adoption of BI screening and referral protocols for IPV survivors. Using a modified Delphi approach and community-academic partnership with CBOs administrators and staff, we identified seven strategies that were considered both generalizable across all CBOs and rated as high priority to be implemented in their organizations. There are a number of promising outcomes of this collaborative project that include: (1) methods for an efficient approach to practically reduce ERIC strategies [36] down to those that have the highest likelihood of achieving broad impact, (2) suggestions for adaptation to strategy definitions for non-clinical settings, (3) demonstrating that CBOs are able to identify strategies across a variety of categories, including, but not limited to, training, and (4) there are still opportunities to better conceptualize dissemination and implementation strategies in CBOs outside of healthcare.

The range of implementation strategies available to CBOs and other settings that can support the adoption, implementation, and sustainment of new evidence-based approaches can be overwhelming [41]. In addition, methods used to support the co-design or co-selection of strategies in partnership with organizational leaders and implementation teams focused on a new intervention can also be time consuming and impractical for community or clinical organizations to fit within limited time and resource constraints [42]. The use of the community-academic partnership to cull ERIC strategies from 73 to 47 greatly reduced the time needed to review strategies in the larger CAB. Further, the use of the CAB and facilitated discussions using video conferencing allowed for a relatively rapid reduction of the 46 strategies to 21 that could be reviewed in detail by CBOs staff and administrators. During the CAB discussion, members also expressed their appreciation of the selection scale options of ‘must have’ and ‘nice to have’ to support the prioritization of strategies. Finally, reduction of the 46 to 21 strategies using a survey prioritization approach allowed CBO staff to complete the process when and where it was most convenient for them.

Our process also confirmed what others focusing on dissemination and implementation science outside of clinical settings have encountered with the ERIC strategies. That is, these strategies require modifications for use at the CBO-level, and many are not relevant outside of healthcare settings [43, 44]. Financial strategies focused on consumers or reimbursements were seen as less relevant by the partnership. The seven prioritized strategies reflect consensus among practitioners working in IPV-focused CBOs, emphasizing pragmatic implementation features—such as local adaptation and survivor input—not typically emphasized in more generalized strategy sets such as ERIC. This illustrates how Delphi methods can surface implementation strategies that are both feasible and relevant within unique service contexts. Further, the prioritization of strategies like training, protocol development, and tailoring reflects PRISM-identified barriers and facilitators at both organizational and infrastructure levels. For example, limited screening capacity and lack of workflows aligned with BI screening and referral protocols underscore the need for structured implementation planning. The inclusion of survivor feedback and promotion of adaptability directly addresses the recipient and context domains of PRISM, signaling the value of community-defined fit over top-down approaches. The ongoing project will also provide opportunities for each CBOs to create additional strategies that may be more relevant locally while also being generalizable to other similar settings.

## Strengths and Limitations

There were some limitations in the strategy selection process. First, community partners that are engaged in this process are all from one region in the Midwest which may not be generalizable to other locations. However, it is likely that IPV-serving CBOs have similar intake, assessment, and care processes regardless of location. Second, we used a convenience sample of 12 CBOs that identified BI screening and referral as an organizational priority which, may limit the generalizability of our findings. This is a consistent challenge in community-engaged research—the focus of the project is on locally identified priorities that may not be shared by other similar organizations. Third, the identification process used in this trial involves the identification of strategies that are likely to support sustained implementation. This is the first phase of our project and does not allow us to describe the actual utility of these strategies until they are tested.

The next steps for this project include a stepped-wedge design whereby we will test a process of CBOs refinement of which strategies, and the order, will be used, operationalized, and enacted. Through this approach, we will provide additional opportunities for the co-design of defined strategies and co-creation of potential new strategies that will increase the reach of BI screening and referral for survivors of IPV.

## Conclusions

This is the first known study to examine processes to select locally relevant implementation strategies for community implementation of BI screening and referral for IPV survivors. The modified Delphi approach appeared to be amenable to CBOs leadership and staff and allowed the reduction of strategies to those that are most needed and likely to have an impact on BI screening implementation. The identified strategies offer valuable insights for optimizing support to IPV survivors and advancing the implementation of public health interventions.

Stakeholder-derived strategies that support comprehensive BI screening and referral in this vulnerable population and timely neurorehabilitation interventions have the potential to mitigate poor health outcomes and identify methods to achieve secondary prevention.

## Supplementary Material

Supplementary Files

This is a list of supplementary files associated with this preprint. Click to download.

• Supplementaryfile1.docx

## Figures and Tables

**Table 1.** Characteristics of participants

Variables	Round 1 (n=14)	Round 2 (n=62)
**Sociodemographic characteristics**		
**Gender, n (%)**		
Male	0 (0%)	2 (3%)
Female	14 (100%)	56 (90%)
Gender nonconforming	0 (0%)	3 (5%)
		
**Age**		
19–25 years	4 (29%)	11 (18%)
26–35 years	0 (0%)	9 (15%)
36–45 years	6 (43%)	18 (29%)
46–55 years	4 (29%)	16 (26%)
Over 55 years	0 (0%)	6 (10%)
**Profession Experience**		
< 1 year	1 (7%)	12 (19%)
1–5 years	4 (29%)	20 (32%)
6–15 years	4 (29%)	11 (18%)
16–25 years	5 (36%)	15 (24%)
Above 25 years	0 (0%)	3 (5%)
		
**Education level**		
High school/ GED	0 (0%)	6 (10%)
Trade school	0 (0%)	0 (0%)
Associate degree	3 (21%)	10 (16%)
Some college	3 (21%)	11 (18%)
College degree	4 (29%)	18 (29%)
Post-graduate (Master and above)	4 (29%)	16 (26%)
**Race**		
African American	0 (0%)	3 (5%)
Asian American	0 (0%)	2 (3%)
Mixed- racial background	1 (7%)	1 (2%)
Native American	1 (7%)	1 (0%)
White-Caucasian	12 (86%)	52 (84%)
**Ethnicity**		
Hispanic/ Latinx	2 (14%)	7 (11%)
Not Hispanic/ Latinx	12 (86%)	54 (87%)
Missing	0 (0%)	3 (5%)

**Table 2-:** Strategies considered for applicability with corresponding original and collaboratively adapted strategy definitions for CBO-based BI screening and referral.

Strategy	ERIC Definitions	BI project definitions
		
1. Access new funding	Access new or existing money to facilitate the implementation	Co-designed by community-academic partnership per protocol
2. Alter incentive/allowance structures	Work to incentivize the adoption and implementation of the clinical innovation	Explore the possibility of offering incentives to clients who actively participate in brain injury screening.
3. Alter patient/consumer fees	Create fee structures where patients/consumers pay less for preferred treatments (the clinical innovation) and more for less-preferred treatments	Not relevant to the project
4. Assess for readiness and identify barriers and facilitators	Assess various aspects of an organization to determine its degree of readiness to implement, barriers that may impede implementation, and strengths that can be used in the implementation effort	Assess the readiness of community-based organizations (CBOs) to implement brain injury screening. This includes considering factors like staff expertise, resources, and infrastructure and identifying barriers, such as staff resistance or resource constraints, that may impede the successful rollout
5. Audit and provide feedback	Collect and summarize clinical performance data over a specified time period and give it to clinicians and administrators to monitor, evaluate, and modify provider behavior	Conduct regular audits of brain injury screening practices and provide feedback to community-based organizations (CBOs) to support quality improvement
6. Build a coalition	Recruit and cultivate relationships with partners in the implementation effort	Co-designed by community-academic partnership per protocol
7. Capture and share local knowledge	Capture local knowledge from implementation sites on how implementers and clinicians made something work in their setting and then share it with other sites	Co-designed by community-academic partnership per protocol
8. Centralize technical assistance	Develop and use a centralized system to deliver technical assistance focused on implementation issues	Establish a centralized system to deliver technical assistance for brain injury screening implementation
9. Change accreditation or membership requirements	Strive to alter accreditation standards so that they require or encourage use of the clinical innovation. Work to alter membership organization requirements so that those who want to affiliate with the organization are encouraged or required to use the clinical innovation	Not relevant to the project
10. Change liability laws	Participate in liability reform efforts that make clinicians more willing to deliver the clinical innovation	Not relevant to the project
11. Change physical structure and equipment	Evaluate current configurations and adapt, as needed, the physical structure and/or equipment (e.g., changing the layout of a room, adding equipment) to best accommodate the targeted innovation	Ensure that facilities are physically accessible and welcoming to clients and their families
12. Change record systems	Change records systems to allow better assessment of implementation or clinical outcomes	Not relevant to the project
13. Change service sites	Change the location of clinical service sites to increase access	Co-designed by community-academic partnership per protocol
14. Conduct cyclical small tests of change	Implement changes in a cyclical fashion using small tests of change before taking changes system-wide. Tests of change benefit from systematic measurement, and results of the tests of change are studied for insights on how to do better. This process continues serially over time, and refinement is added with each cycle	Implement periodic changes using small tests of brain injury screening before taking changes system-wide
15. Conduct educational meetings	Hold meetings targeted toward different stakeholder groups (e.g., providers, administrators, other organizational stakeholders, and community, patient/consumer, and family stakeholders) to teach them about the clinical innovation	Host educational meetings or seminars within the organization to inform staff about the importance of brain injury screening and the associated benefits.
16. Conduct educational outreach visits	Have a trained person meet with providers in their practice settings to educate providers about the clinical innovation with the intent of changing the provider’s practice	Conduct outreach visits to other community-based organizations to share knowledge and best practices related to brain injury screening.
17. Conduct local consensus discussions	Include local providers and other stakeholders in discussions that address whether the chosen problem is important and whether the clinical innovation to address it is appropriate	Co-designed by community-academic partnership per protocol
18. Conduct local needs assessment	Collect and analyze data related to the need for the innovation	Not relevant to the project
19. Conduct ongoing training	Plan for and conduct training in the clinical innovation in an ongoing way	Offer continuous training opportunities to staff members to ensure they remain competent in conducting brain injury screenings.
20. Create a learning collaborative	Facilitate the formation of groups of providers or provider organizations and foster a collaborative learning environment to improve implementation of the clinical innovation	Co-designed by community-academic partnership per protocol
21. Create new clinical teams	Change who serves on the clinical team, adding different disciplines and different skills to make it more likely that the clinical innovation is delivered (or is more successfully delivered)	Establish dedicated teams or units specifically focused on brain injury screening and management
22. Create or change credentialing and/or licensure standards	Create an organization that certifies clinicians in the innovation or encourage an existing organization to do so. Change governmental professional certification or licensure requirements to include delivering the innovation. Work to alter continuing education requirements to shape professional practice toward the innovation	Not relevant to the project
23. Develop a formal implementation blueprint	Develop a formal implementation blueprint that includes all goals and strategies. The blueprint should include the following: 1) aim/purpose of the implementation; 2) scope of the change (e.g., what organizational units are affected); 3) timeframe and milestones; and 4) appropriate performance/progress measures. Use and update this plan to guide the implementation effort over time	Develop a detailed implementation plan that outlines the steps, responsibilities, and timelines for implementing brain injury screening throughout the community-based organizations (CBOs)
24. Develop academic partnerships	Partner with a university or academic unit for the purposes of shared training and bringing research skills to an implementation project	Co-designed by community-academic partnership per protocol
25. Develop an implementation glossary	Develop and distribute a list of terms describing the innovation, implementation, and stakeholders in the organizational change	Create a local implementation glossary that defines key terms and concepts specific to brain injury screening and referral
26. Develop and implement tools for quality monitoring	Develop, test, and introduce into quality-monitoring systems the right input–the appropriate language, protocols, algorithms, standards, and measures (of processes, patient/consumer outcomes, and implementation outcomes) that are often specific to the innovation being implemented	Create standardized protocols and tools for quality monitoring of brain injury screening that align with best practices
27. Develop and organize quality monitoring systems	Develop and organize systems and procedures that monitor clinical processes and/or outcomes for the purpose of quality assurance and improvement	Set up an efficient system for tracking the progress of brain injury screening, including data collection, reporting, and analysis
28. Develop disincentives	Provide financial disincentives for failure to implement or use the clinical innovations	Not relevant to the project
29. Develop educational materials	Develop and format manuals, toolkits, and other supporting materials in ways that make it easier for stakeholders to learn about the innovation and for clinicians to learn how to deliver the clinical innovation	Create locally tailored educational materials that explain the purpose and process of brain injury screening.
30. Develop resource sharing agreements	Develop partnerships with organizations that have resources needed to implement the innovation	Collaborate with neighboring facilities to establish resource-sharing agreements that facilitate the exchange of expertise, equipment, or personnel
31. Distribute educational materials	Distribute educational materials (including guidelines, manuals, and toolkits) in person, by mail, and/or electronically	Disseminate educational materials to staff at community-based organizations, clients and their families through various channels, including reception area, websites, and community events.
32. Facilitate relay of clinical data to providers	Provide as close to real-time data as possible about key measures of process/outcomes using integrated modes/channels of communication in a way that promotes use of the targeted innovation	Develop clear communication strategies to convey the potential risks and consequences of not participating in brain injury screening
33. Facilitation	A process of interactive problem solving and support that occurs in a context of a recognized need for improvement and a supportive interpersonal relationship	Employ facilitators who can help organizational teams navigate the complexities of brain injury screening implementation.
34. Fund and contract for the clinical innovation	Governments and other payers of services issue requests for proposals to deliver the innovation, use contracting processes to motivate providers to deliver the clinical innovation, and develop new funding formulas that make it more likely that providers will deliver the innovation	Not relevant to the project
35. Identify and prepare champions	Identify and prepare individuals who dedicate themselves to supporting, marketing, and driving through an implementation, overcoming indifference or resistance that the intervention may provoke in an organization	Identify individuals who are passionate about the importance of brain injury screening and willing to champion the cause.
36. Identify early adopters	Identify early adopters at the local site to learn from their experiences with the practice innovation	Identify and engage staff within the community-based organizations (CBOs) who are enthusiastic about implementing brain injury screening.
37. Increase demand	Attempt to influence the market for the clinical innovation to increase competition intensity and to increase the maturity of the market for the clinical innovation	Not relevant to the project
38. Inform local opinion leaders	Inform providers identified by colleagues as opinion leaders or “educationally influential” about the clinical innovation in the hopes that they will influence colleagues to adopt it	Identify and engage local opinion leaders and influential figures within the community who can help promote the benefits of brain injury screening
39. Intervene with patients/consumers to enhance uptake and adherence	Develop strategies with patients to encourage and problem solve around adherence	Develop client support programs to address barriers to uptake and adherence, such as transportation issues or language barriers
40. Involve executive boards	Involve existing governing structures (e.g., boards of directors, medical staff boards of governance) in the implementation effort, including the review of data on implementation processes	Not relevant to the project
41. Involve patients/consumers and family members	Engage or include patients/consumers and families in the implementation effort	Engage clients and their family members as partners in the brain injury screening process from the outset
42. Make billing easier	Make it easier to bill for the clinical innovation	Not relevant to the project
43. Make training dynamic	Vary the information delivery methods to cater to different learning styles and work contexts, and shape the training in the innovation to be interactive	Use engaging and interactive training methods that encourage active participation and knowledge retention.
44. Mandate change	Have leadership declare the priority of the innovation and their determination to have it implemented	Collaborate with regulators or policymakers to establish mandates or guidelines that require the integration of brain injury screening into standard practice for DVSA organizations.
45. Model and simulate change	Model or simulate the change that will be implemented prior to implementation	Use simulation tools to illustrate the potential impact of brain injury screening on patient outcomes.
46. Obtain and use patients/consumers and family feedback	Develop strategies to increase patient/consumer and family feedback on the implementation effort	Actively seek feedback from survivors of intimate partner violence regarding their experiences with brain injury screening
47. Obtain formal commitments	Obtain written commitments from key partners that state what they will do to implement the innovation	Co-designed by community-academic partnership per protocol
48. Organize clinician implementation team meetings	Develop and support teams of clinicians who are implementing the innovation and give them protected time to reflect on the implementation effort, share lessons learned, and support one another’s learning	Schedule regular meetings of implementation teams to share progress, exchange ideas, and address challenges.
49. Place innovation on fee for service lists/formularies	Work to place the clinical innovation on lists of actions for which providers can be reimbursed (e.g., a drug is placed on a formulary, a procedure is now reimbursable)	Not relevant to the project
50. Prepare patients/consumers to be active participants	Prepare patients/consumers to be active in their care, to ask questions, and specifically to inquire about care guidelines, the evidence behind clinical decisions, or about available evidence-supported treatments	Educate clients about the importance of their active involvement in the screening process.
51. Promote adaptability	Identify the ways a clinical innovation can be tailored to meet local needs and clarify which elements of the innovation must be maintained to preserve fidelity	Encourage adaptability within the implementation process, allowing community-based organizations (CBOs) teams to tailor the screening approach to individual clients needs and settings
52. Promote network weaving	Identify and build on existing high-quality working relationships and networks within and outside the organization, organizational units, teams, etc. to promote information sharing, collaborative problem-solving, and a shared vision/goal related to implementing the innovation	Facilitate networking and collaboration among different community-based organizations (CBOs), departments, and organizations involved in brain injury screening
53. Provide clinical supervision	Provide clinicians with ongoing supervision focusing on the innovation. Provide training for clinical supervisors who will supervise clinicians who provide the innovation	Implement a system of supervision to oversee the quality and consistency of brain injury screening
54. Provide local technical assistance	Develop and use a system to deliver technical assistance focused on implementation issues using local personnel	Establish local technical assistance teams within community-based organizations (CBOs) to provide on-site support and guidance during the implementation of brain injury screening
55. Provide ongoing consultation	Provide ongoing consultation with one or more experts in the strategies used to support implementing the innovation	Offer ongoing consultation and support to organization teams as they encounter challenges during the implementation of brain injury screening.
56. Purposely reexamine the implementation	Monitor progress and adjust clinical practices and implementation strategies to continuously improve the quality of care	Monitor progress and adjust community-based organizations (CBOs) practices and implementation strategies to continuously improve the quality of brain injury screening implementation
57. Recruit, designate, and train for leadership	Recruit, designate, and train leaders for the change effort	Identify and train local leaders who can assume leadership roles within the implementation teams
58. Remind clinicians	Develop reminder systems designed to help clinicians to recall information and/or prompt them to use the clinical innovation	Implement regular reminders and notifications within the organization to conduct brain injury screenings when appropriate.
59. Revise professional roles	Shift and revise roles among professionals who provide care, and redesign job characteristics	Redefine the roles and responsibilities of community-based organizations (CBOs) staff to incorporate brain injury screening as a standard practice
60. Shadow other experts	Provide ways for key individuals to directly observe experienced people engage with or use the targeted practice change/innovation	Arrange for staff to shadow experienced experts in brain injury screening to gain hands-on experience and learn best practices.
61. Stage implementation scale up	Phase implementation efforts by starting with small pilots or demonstration projects and gradually move to a system wide rollout	Phase implementation efforts by starting with small pilots or demonstration projects and gradually move to a system wide rollout
62. Start a dissemination organization	Identify or start a separate organization that is responsible for disseminating the clinical innovation. It could be a for-profit or non-profit organization	Not relevant to the project
63. Tailor strategies	Tailor the implementation strategies to address barriers and leverage facilitators that were identified through earlier data collection	Customize implementation strategies to align with the unique characteristics of the community-based organizations (CBOs), client population, and local culture
64. Use advisory boards and workgroups	Create and engage a formal group of multiple kinds of stakeholders to provide input and advice on implementation efforts and to elicit recommendations for improvements	Establish local advisory boards or workgroups composed of diverse stakeholders to provide input and guidance on the implementation process
65. Use an implementation advisor	Seek guidance from experts in implementation	Seek guidance from an implementation advisor with expertise in brain injury screening.
66. Use capitated payments	Pay providers or care systems a set amount per patient/consumer for delivering clinical care	Not relevant to the project
67. Use data experts	Involve, hire, and/or consult experts to inform management on the use of data generated by implementation efforts	Co-designed by community-academic partnership per protocol
68. Use data warehousing techniques	Integrate clinical records across facilities and organizations to facilitate implementation across systems	Implement data warehousing techniques to efficiently store, manage, and analyze large volumes of screening data
69. Use mass media	Use media to reach large numbers of people to spread the word about the clinical innovation	Not relevant to the project
70. Use other payment schemes	Introduce payment approaches (in a catch-all category)	Not relevant to the project
71. Use train-the-trainer strategies	Train designated clinicians or organizations to train others in the clinical innovation	Train a select group within community-based organizations as trainers who can then educate their peers on the principles and practices of brain injury screening.
72. Visit other sites	Visit sites where a similar implementation effort has been considered successful	Visit other organizations that have successfully implemented brain injury screening.
73. Work with educational institutions	Encourage educational institutions to train clinicians in the innovation	Not relevant to the project

**Table 3.** List of strategies rated in Round 1 and Round 2

		ROUND 1 (n=9)				ROUND 2 (n=55)		
	MUST HAVE	NICE TO HAVE	DON’T NEED	NEED MORE INFO	MUST HAVE	NICE TO HAVE	DON’T NEED	NEED MORE INFO
EDUCATE STRATEGIES								
1- Develop an implementation glossary	44%	33%	0%	22%				
2- Recruit, designate, and train for leadership	89%	0%	0%	11%	45%	35%	15%	5%
3-Visit other sites	22%	33%	33%	11%				
4-Conduct ongoing training.	78%	22%	0%	0%	64%	76%	0%	2%
5- Conduct educational meetings.	44%	56%	0%	0%				
6- Conduct educational outreach visits.	22%	44%	33%	0%				
7- Develop educational materials.	67%	22%	0%	11%				
8- Distribute educational materials.	22%	33%	22%	22%				
9- Make training dynamic	78%	0%	11%	11%				
10- Shadow other experts	11%	56%	22%	11%				
11- Use train-the-trainer strategies	33%	71%	0%	11%	29%	56%	44%	44%
12-Prepare patients/consumers to be active participants	44%	44%	0%	11%	55%	31%	33%	56%
		ROUND 1 (n=5)				ROUND 2(n=55)		
TECHNICAL ASSISTANCE STRATEGIES	MUST HAVE	NICE TO HAVE	DON’T NEED	NEED MORE INFO	MUST HAVE	NICE TO HAVE	DON’T NEED	NEED MORE INFO
13- Centralize technical assistance	40%	20%	40%	0%				
14- Facilitation	20%	60%	20%	0%				
15- Provide local technical assistance	40%	0%	20%	40%				
16-Use an implementation adviser	40%	60%	0%	0%	49%	45%	2%	4%
17- Provide ongoing consultation	80%	20%	0%	0%	73%	27%	0%	0%
		ROUND 1 (n=5)				ROUND 2(n=54)		
BUILD STAFF AND LEADERSHIP STRATEGIES	MUST HAVE	NICE TO HAVE	DON’T NEED	NEED MORE INFO	MUST HAVE	NICE TO HAVE	DON’T NEED	NEED MORE INFO
18- Assess for readiness and identify barriers and facilitators	80%	20%	0%	0%	52%	39%	6%	4%
19- Develop a formal implementation blueprint	100%	0%	0%	0%	76%	22%	2%	0%
20-Identify early adopters.	40%	60%	0%	0%	43%		6%	2%
21- Identify and prepare champions.	20%	80%	0%	0%				
22- Inform local opinion leaders	0%	60%	20%	20%				
23- Organize clinician implementation team meetings.	100%	20%	20%	0%	33%	61%	4%	2%
24-Promote network weaving	0%	60%	20%	20%				
25- Use advisory boards and workgroups	0%	40%	20%	40%				
26 Involve patients/consumers and family members	60%	20%	0%	20%	43%	37%	7%	13%
		ROUND 1 (n=5)				ROUND 2(n=54)		
MANAGEMENT AND EVALUATION STRATEGIES	MUST HAVE	NICE TO HAVE	DON’T NEED	NEED MORE INFO	MUST HAVE	NICE TO HAVE	DON’T NEED	NEED MORE INFO
27-Audit and provide feedback	60%	20%	0%	20%	24%	65%	7%	4%
28-Develop and implement tools for quality monitoring	80%	20%	0%	0%	70%	22%	4%	4%
29-Develop and organize quality monitoring systems	40%	60%	0%	0%				
30-Purposely reexamine the implementation	60%	40%	0%	0%	46%	48%	2%	4%
31- Obtain and use patients/consumers and family feedback	60%	40%	0%	0%	65%	30%	6%	0%
32- Provide clinical supervision	0%	60%	0%	40%				
33- Use data warehousing techniques	40%	40%	0%	20%				
34- Conduct cyclical small tests of change	20%	40%	0%	40%				
35-Stage implementation scale up	20%	60%	0%	20%				
36- Model and simulate change.	0%	40%	20%	40%				
		ROUND 1(n=4)				ROUND 2 (n=44)		
ORGANIZATIONAL AND WORKFLOW STRATEGIES	MUST HAVE	NICE TO HAVE	DON’T NEED	NEED MORE INFO	MUST HAVE	NICE TO HAVE	DON’T NEED	NEED MORE INFO
37-Promote adaptability	75%	25%	0%	0%	75%	20%	2%	2%
38- Tailor strategies	75%	25%	0%	0%	68%	23%	7%	2%
39- Create new clinical teams	0%	50%	0%	50%				
40- Develop resource sharing agreements	0%	75%	0%	25%				
41- Remind clinicians	50%	50%	0%	0%	27%	59%	11%	2%
42- Revise professional roles	50%	50%	0%	0%	25%	57%	5%	14%
43- Intervene with patients/consumers to enhance uptake and adherence	75%	25%	0%	0%	45%	45%	2%	7%
44- Facilitate relay of clinical data to providers	25%	25%	25%	25%				
45- Change physical structure and equipment	75%	0%	25%	0%	57%	32%	5%	7%
46-Alter incentive/allowance structures.	25%	25%	50%	0%				
47-Mandate change	0%	25%	25%	50%				

**Table 4.** List of strategies included in Round 2

Educate Strategies	• Identify and train local leaders who can assume leadership roles within the implementation teams • Offer continuous training opportunities to staff members, including the creation of locally tailored educational materials and the use of engaging, interactive training methods to ensure they remain competent in conducting brain injury screenings (combination of strategies 4,7,9 from the original survey) • Train a select group within community-based organizations as trainers who can then educate their peers on the principles and practices of brain injury screening • Educate clients about the importance of their active involvement in the screening process.
Build Staff & Leadership Strategies	• Assess the readiness of community-based organizations (CBOs) to implement brain injury screening. This includes considering factors like staff expertise, resources, and infrastructure and identifying barriers, such as staff resistance or resource constraints, that may impede the successful rollout • Develop a detailed implementation plan that outlines the steps, responsibilities, and timelines for implementing brain injury screening throughout the community-based organizations (CBOs) • Identify and engage passionate individuals within community-based organizations (CBOs) who are enthusiastic about implementing and championing the importance of brain injury screening and referral (combination of strategies 20 and 21 and from the original survey). • Schedule regular meetings of implementation teams to share progress, exchange ideas, and address challenges. • Engage clients and their family members as partners in the brain injury screening process from the outset
Technical Assistance Strategies	• Seek guidance from an implementation advisor with expertise in brain injury screening. • Offer ongoing consultation and support to organization teams as they encounter challenges during the implementation of brain injury screening.
Management And Evaluation Strategies	• Conduct regular audits of brain injury screening practices and provide feedback to community-based organizations (CBOs) to support quality improvement • Create standardized protocols and tools for quality monitoring of brain injury screening that align with best practices • Monitor progress and adjust community-based organizations (CBOs) practices and implementation strategies to continuously improve the quality of brain injury screening implementation • Actively seek feedback from survivors of intimate partner violence regarding their experiences with brain injury screening
Organizational And Workflow Strategies	• Encourage adaptability within the implementation process, allowing community-based organizations (CBOs) teams to tailor the screening approach to individual patient needs and settings • Customize implementation strategies to align with the unique characteristics of the community-based organizations (CBOs), patient population, and local culture • Implement regular reminders and notifications within the organization to conduct brain injury screenings when appropriate. • Redefine the roles and responsibilities of community-based organizations (CBOs) staff to incorporate brain injury screening as a standard practice • Develop client support programs to address barriers to uptake and adherence, such as transportation issues or language barriers • Ensure that facilities are physically accessible and welcoming to clients and their families

**Table 5.** Final list of strategies

Educate Strategies	• Offer continuous training opportunities to staff members, including the creation of locally tailored educational materials and the use of engaging, interactive training methods to ensure they remain competent in conducting brain injury screenings.
Build Staff & Leadership Strategies	• Develop a detailed implementation plan that outlines the steps, responsibilities, and timelines for implementing brain injury screening throughout the community-based organizations (CBOs)
Technical Assistance Strategies	• Offer ongoing consultation and support to organization teams as they encounter challenges during the implementation of brain injury screening.
Management And Evaluation Strategies	• Create standardized protocols and tools for quality monitoring of brain injury screening that align with best practices • Actively seek feedback from survivors of intimate partner violence regarding their experiences with brain injury screening
Organizational And Workflow Strategies	• Encourage adaptability within the implementation process, allowing community-based organizations (CBOs) teams to tailor the screening approach to individual clients needs and settings • Customize implementation strategies to align with the unique characteristics of the community-based organizations (CBOs), client population, and local culture

## Data Availability

The datasets used and/or analyzed during the current study are available from the corresponding author on reasonable request.

## Abbreviations

BI: Brain Injury
CAB: Community-campus advisory board
CBOs: Community-based organizations
ERIC: Expert Recommendations for Implementing Change
IPV: Intimate partner violence
TBI: Traumatic Brain Injury
